# Differentiating injury patterns and outcomes in accidental, suicidal and occupational falls from heights

**DOI:** 10.1007/s00068-026-03165-w

**Published:** 2026-04-30

**Authors:** Christoph Beyersdorf, Bjoern Hussmann, Niklas Wergen, Rolf Lefering, Erik Schiffner, Uwe Maus, Carina Jaekel, TraumaRegister DGU

**Affiliations:** 1 Department of Orthopaedics and Trauma Surgery, Faculty of Medicine, University Hospital Duesseldorf, Heinrich-Heine- University Duesseldorf, 40225 Duesseldorf, Germany; 2 Department of Trauma and Orthopedics Surgery, Alexianer Hospital Hochsauerland, Petriweg 2, 59759 Arnsberg- Hüsten, Germany; 3 Institute for Research in Operative Medicine (IFOM), University Witten/Herdecke, 58455 Cologne, Germany; 4 Committee on Emergency Medicine, Intensive Care and Trauma Management (Sektion NIS) of the German Trauma Society (DGU), 10623 Berlin, Germany

**Keywords:** Falls, Accidental, Suicidal, Work-related, Traumaregister

## Abstract

**Background:**

Falls from significant heights are a leading cause of severe injuries and can result from accidents, suicide attempts, or occupational incidents. While previous studies have explored general injury patterns associated with falls, little is known about the specific differences between accidental, suicidal, and occupational falls. This study aims to identify distinct injury patterns and outcomes among these fall types, thereby enhancing primary care and decision-making algorithms.

**Methods:**

We conducted a retrospective analysis using data from the German TraumaRegister DGU^®^ (2014–2023), focusing on patients who experienced falls from heights. Only patients admitted to trauma centers with trauma team activation and a Maximum Abbreviated Injury Scale (MAIS) severity ≥ 3 were included. All falls were categorized in accidental, suicidal, or occupational and distinguished between high (≥ 3 m) and low (< 3 m) falls. Statistical analyses included chi-squared tests for categorical variables and Mann-Whitney U tests for continuous variables, with significance set at *p* < 0.05.

**Results:**

Among 188,708 patients, 29,991 (15.9%) experienced high falls, while 50,674 (26.9%) fell from low height. High falls were predominantly accidental (82.3%), with suicidal falls accounting for 17.7%. Accidental high falls mainly caused thoracic (58.9%) and head injuries (46.2%), whereas suicidal falls frequently involved pelvic (55.7%) and lower limb injuries (56.5%). Suicidal falls resulted in significantly higher injury severity (ISS 30.6 vs. 21.4 in accidents, *p* < 0.001) and mortality (25.4% vs. 11.0%, *p* < 0.001). Occupational falls, predominantly affecting men, demonstrated a lower mortality rate (6.5%) compared to non-occupational high falls (16.1%).

**Conclusion:**

This study reveals that suicidal falls from height lead to more severe injuries and higher mortality compared to accidental or occupational falls. Accidental falls are more associated with head trauma, while suicidal falls primarily involve the lower extremities and pelvis. Occupational falls have a lower mortality rate, possibly due to faster medical intervention. Understanding these patterns is crucial for tailoring early clinical management and improving patient outcomes.

## Introduction

Falls from significant heights are a common cause of severe injuries and can occur accidentally, occupationally, or as an act of suicide. Among self-inflicted injuries, falls are the most prevalent mechanism [1]. In the United States of America, fall-related trauma is also the leading cause of fatalities among construction workers [2].

Mortality and morbidity associated with falls from great heights remains considerable. More than 50% of patients require intensive care treatment after falling from six meters [3]. A fall from ≥ 3 m is still considered an indication for trauma room management, as confirmed by the 2022 revision of the German polytrauma guideline [4]. Currently, computed tomography (CT)-based imaging is recommended for falls exceeding four meters. However, little is known about the specific injury patterns following falls and how they differ between accidental, suicidal, and occupational falls.

Accident kinematics and clinical findings are integral to the initial clinical decision-making process. For instance, a fall from a height of 10 m can result in an impact velocity of approximately 50 km/h. High-energy trauma, such as falls from great heights, combined with clinical suspicion leads to unstable pelvic injuries in 50% of cases [5]. While geriatric patients are more likely to suffer traumatic brain injuries and coagulopathies after low-height falls, the prognostic determinants and clinical courses of falls from greater heights remain less understood [6].

Because polytraumatized patients often rely on external sources for their medical history, which are transferred from the prehospital to the clinical phase, the assessment of injury severity frequently depends heavily on accident kinematics. In addition to classifying an event as high-energy trauma, which includes falls from great heights, further differentiation of the causes of falls is currently not feasible. However, such differentiation of falls based on circumstances- suicidal, accidental or occupational- can facilitate prognostic assessment and improve the clinical decision-making algorithm.

Preliminary data suggest that suicidal falls often result in feet-first landings, whereas accidental falls frequently involve uncoordinated impacts, leading to a higher prevalence of traumatic brain and thoracic injuries and increased mortality rates [7]. Nonetheless, the impact of these differences on early clinical management remains unclear. Similarly, no comprehensive data currently exists on occupational falls, representing another gap in knowledge.

This study aims to differentiate the injury patterns and severity among patients who have experienced accidental, suicidal, or occupational falls from varying heights. Additionally, it seeks to document primary care interventions and their impact on outcomes for each patient group. The goal is to enhance awareness during initial care about specific injury patterns and effective therapeutic measures tailored to each group, facilitating timely and appropriate interventions.

We hypothesize that suicidal falls produce distinct injury patterns compared to accidental or occupational falls. A retrospective analysis of trauma data of the German TraumaRegister DGU^®^ may reveal that fall height and intent are significant factors influencing the type and severity of injuries as well as clinical outcomes. Such insights hold critical relevance for primary care teams, enabling the anticipation of common injury patterns and better preparation for effective interventions.

## Materials and methods

The German TraumaRegister DGU^®^ (TR-DGU) was founded in 1993. The aim of this multicenter database is a pseudonymized and standardized documentation of severely injured patients.

Data are collected prospectively in four consecutive time phases from the site of the accident until discharge from hospital: (A) Pre-hospital phase, (B) Emergency room and initial surgery, (C) Intensive care unit and (D) Discharge. The documentation includes detailed information on demographics, injury pattern, comorbidities, pre- and in-hospital management, course on intensive care unit, relevant laboratory findings including data on transfusion and outcome of each individual. The inclusion criterion is admission to hospital via emergency room with subsequent ICU/ICM care or reach the hospital with vital signs and die before admission to ICU. The infrastructure for documentation, data management, and data analysis is provided by AUC – Academy for Trauma Surgery (AUC - Akademie der Unfallchirurgie GmbH), a company affiliated to the German Trauma Society. The scientific leadership is provided by the Committee on Emergency Medicine, Intensive Care and Trauma Management (Sektion NIS) of the German Trauma Society. The participating hospitals submit their data pseudonymized into a central database via a web-based application. Scientific data analysis is approved according to a peer review procedure laid down in the publication guideline of TraumaRegister DGU^®^. The participating hospitals are primarily located in Germany (90%), but a rising number of hospitals of other countries contribute data as well (at the moment from Austria, Belgium, China, Finland, Luxembourg, Slovenia, Switzerland, The Netherlands, and the United Arab Emirates). Currently, over 35,000 cases from more than 700 hospitals are entered into the database per year. Participation in TR-DGU is voluntary. For hospitals associated with TraumaNetzwerk DGU^®^, however, the entry of at least a basic data set is obligatory for reasons of quality assurance. We analyzed the 2014–2023 TR-DGU dataset.

### Patients

Trauma patients primarily admitted to hospitals in Germany, Austria or Switzerland in 2014–2023 qualified for analysis. Only patients with trauma team activation and Maximum Abbreviated Injury Scale (MAIS) severity ≥ 3 were included. Patients transferred out early (< 48 h) were excluded since the final outcome was missing. Patients transferred in from other hospitals were also excluded due to the limited information about prehospital and initial clinical management. Within this patient group specific analyses were performed for the high fall (≥ 3 m) and low fall (< 3 m) subgroups. In case of a missing cause of accident (2.1% of cases), or if the cause was violence / assault (0.3% in falls), patients were excluded from analysis. Information about work-related or leisure-related accidents was available only since the 2020 revision of the registry dataset.

### Statistics

Descriptive analysis was performed with SPSS (version 29, IBM Corp., Armonk, NY, United States). Numbers of cases, percentages, means, and standard deviations (SD) were reported for normally distributed data. In case of non-normally distributed (skewed) data, median values with interquartile ranges (IQR) were reported. The chi-squared test was used for categorical variables, the Mann-Whitney U test was used for metric variables. A p-value < 0.05 was considered statistically significant. The data is presented using Prism 8.0 (GraphPad, La Jolla, CA, United States) and Excel (Microsoft, Redmond, WA, United States) software.

The study was performed in accordance with the publication guideline of the TraumaRegister DGU^®^ and is registered as TR-DGU Project ID 2024-013. Since the study was a retrospective anonymized analysis, ethical approval was not required according to the regulations of the responsible regional medical association. The authors had no access to information that could identify individual participants during or after data collection. Anonymous data was accessed on August 14th 2024.

## Results

**Fig. 1:**
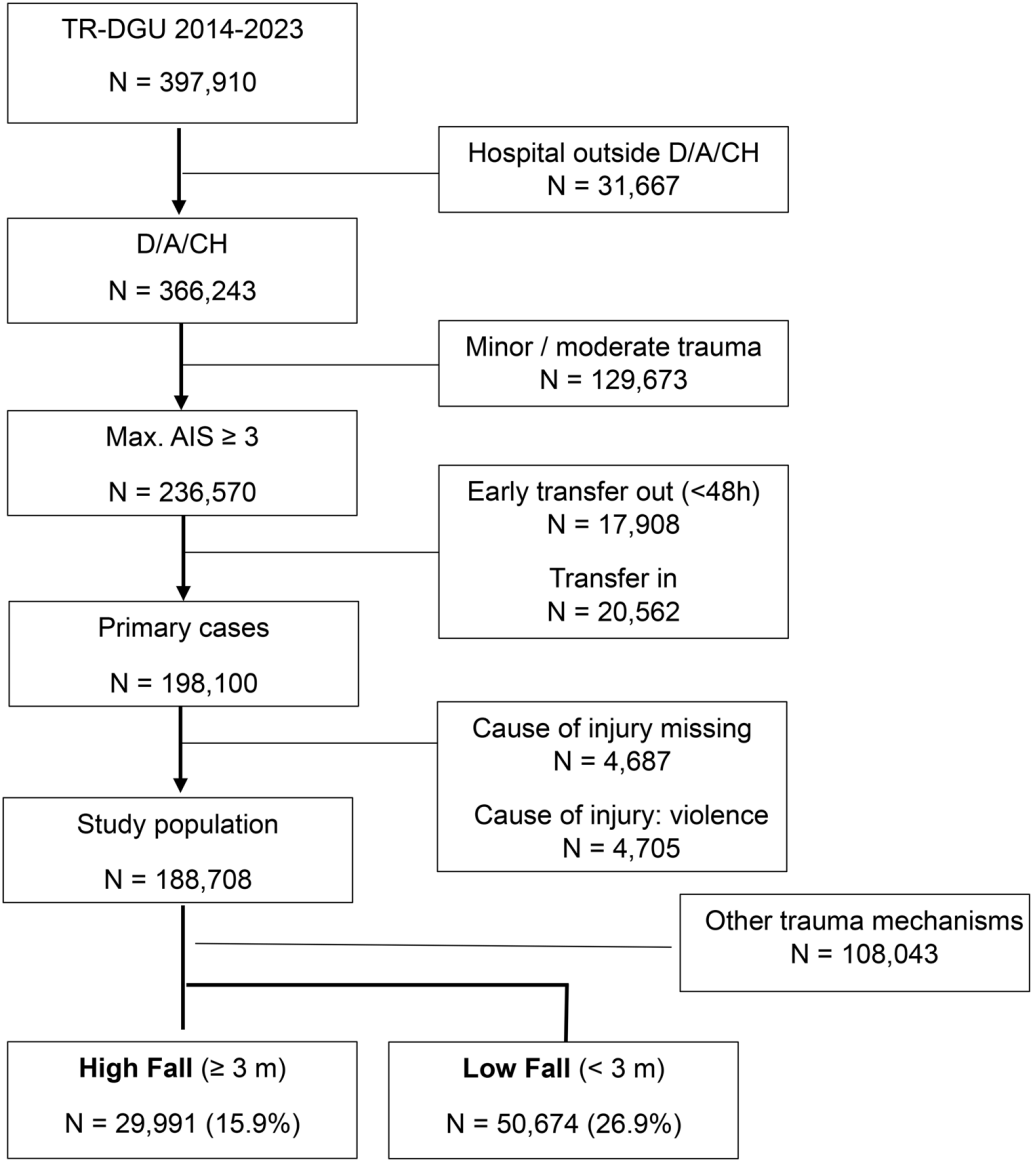
Cohort identification. D: Germany, A: Austria, CH: Switzerland, AIS: Abbreviated Injury Score

After applying the inclusion and exclusion criteria shown in Fig. 1, a total of 188,808 patients were identified for inclusion. Since 2014, falls have been responsible for 41.7% of the cases included in the TR-DGU (*n* = 82,678). As described above, cases with a missing cause or where the cause was violence/assault are excluded from further analysis (*n* = 82,665). These data were collected from a total of 748 hospitals. A distinction is made between low falls from a height of < 3 m (*n* = 50,674), and high falls from a height ≥ 3 m (*n* = 29,991, 37.2%; Fig. 1).

**Fig. 2:**
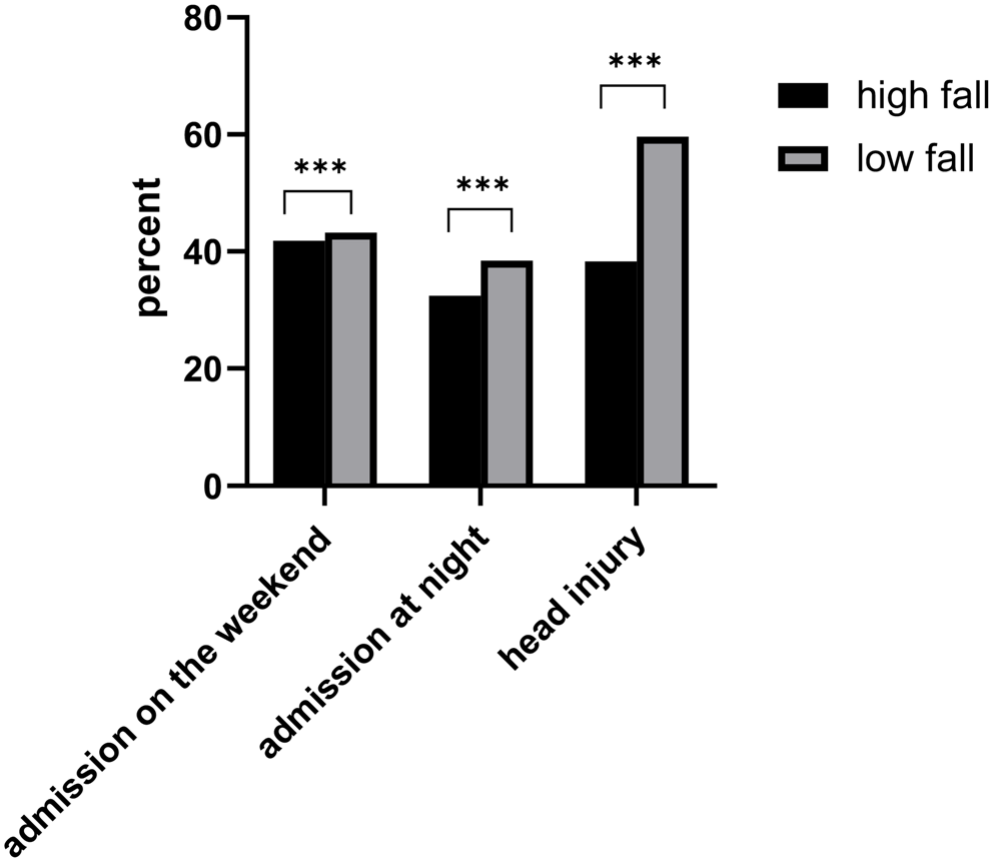
Comparison of the specified parameters between high and low. The data are presented as percentages of the respective group involved

Of these high falls, 82.3% (*n* = 24,686) were accidents, while 17.7% (*n* = 5,305) were (suspected) suicide attempts. The mean age of the high falls group was 53.6 years (SD 20.5) for the accidental and 46.3 (SD 19.8) years for the suspected suicide group, while the low falls group represented the oldest cohort among all mechanisms, with a mean age of 69.6 years (SD 18.8, Fig. 2). The Injury Severity Score (ISS) in the accidental high falls group averaged 21.4 (SD 10.9), in the suicidal high falls group 30.6 (SD 15.1; 23.0 (SD 12.3) across all high falls patients) and in the low falls group, it was 18.7 (SD 9.1; see Table 1).

**Table 1:** Patient demographics and clinical parameters

	Accidental high falls (*n* = 24,686)		Suicidal high falls (*n* = 5,305)		Low falls (*n* = 50,674)	
***General data***						
Age (mean)	53.6	SD 20.5	46.3	SD 19.8	69.6	SD 18.8
ISS (mean)	21.4	SD 10.9	30.6	SD 15.1	18.7	SD 9.1
Male sex (n)	20,607	83.5%	3,059	57.5%	29,037	57.3%
***Prehospital / Emergency room***						
Prehospital time (minutes)	62	IQR 49–80	55	IQR 42–72	62	IQR 48–80
Resuscitation (n)	635	2.7%	517	10.0%	1,152	2.4%
Intubation (n)	5,128	21.5%	2,059	39.6%	9,527	20.1%
Emergency surgery (n)	6,072	24.6%	2,466	46.5%	7,455	14.7%
Blood transfusion (n)	1,621	6.6%	1,552	29.9%	1,793	3.5%
***Trauma care level***						
Supra-regional (n)	16,425	66.5%	3,985	75.1%	28,581	56.4%
Regional (n)	6,659	27.0%	1,137	21.4%	17,420	34.5%
Local (n)	1,602	6.5%	183	3.4%	4,623	9.1%
***Clinical course***						
ICU Length of stay (days)	2	IQR 1–7	11	IQR 5–22	2	IQR 1–6
Hospital Length of stay (days)	13	IQR 8–22	24	IQR 14–39	10	IQR 5–17
In-Hospital mortality (n)	2,721	11.0%	1,348	25.4%	11,443	22.6%
Mortality within 24 h (n)	1,350	5.5%	944	17.8%	5,154	10.2%

SD: Standard deviation; IQR: Interquartile range

Significantly more men were affected by high falls (78.9% vs. 57.3% in the low falls group, *p* < 0.001). More patients died in the hospital following a low fall (22.6% vs. 13.6% for high falls, *p* < 0.001). Additionally, head injuries were significantly more frequent in the low falls group (59.6%) compared to the high falls group (38.1%, *p* < 0.001).

Slight differences regarding the date and time of admission were also observed: 41.8% of high fall cases and 43.2% of low fall cases were admitted during the weekend (Friday–Sunday, *p* < 0.001). Nighttime admissions were slightly more common in the low falls group (38.4%) than in the high falls group (32.2%, *p* < 0,001; Fig. 2).

### Comparison of accidental and suicidal high falls

**Fig. 3:**
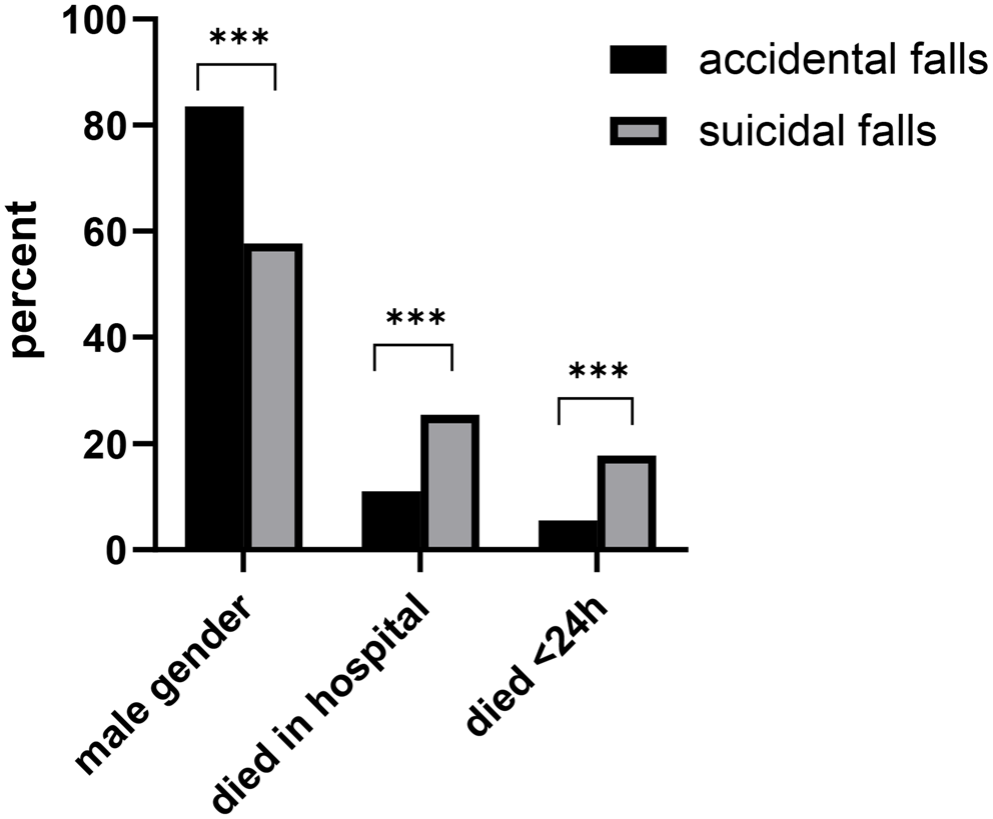
Comparison of the indicated categories between accidents and suicides in the high falls group. The data are presented as percentages of the respective group involved

Next, we compared suicidal cases and accidental trauma within the category of high falls. Patients with accidental injuries were slightly older, with a mean age of 53.6 years, compared to suicidal patients (46.3 years, *p* < 0.001) and 83.5% (*n* = 20,607) of patients after accidents and 57.5% of patients after suicidal attempts (*n* = 3,056) were male (*p* < 0.001). Suicidal patients were significantly more severely injured, with an ISS of 30.6 compared to 21.4 for accidents (*p* < 0.001). This was also reflected by the mortality rates: 25.4% (*n* = 1,348) of suicidal cases died in the hospital, compared to 11.0% (*n* = 2,721) of accidental cases (*p* < 0.001). Among those who died in the hospital, 70.0% of suicidal cases (*n* = 944) and 49.6% (*n* = 1,350) of accidental cases passed away within the first 24 h (*p* < 0.001; Fig. 3).

**Fig. 4:**
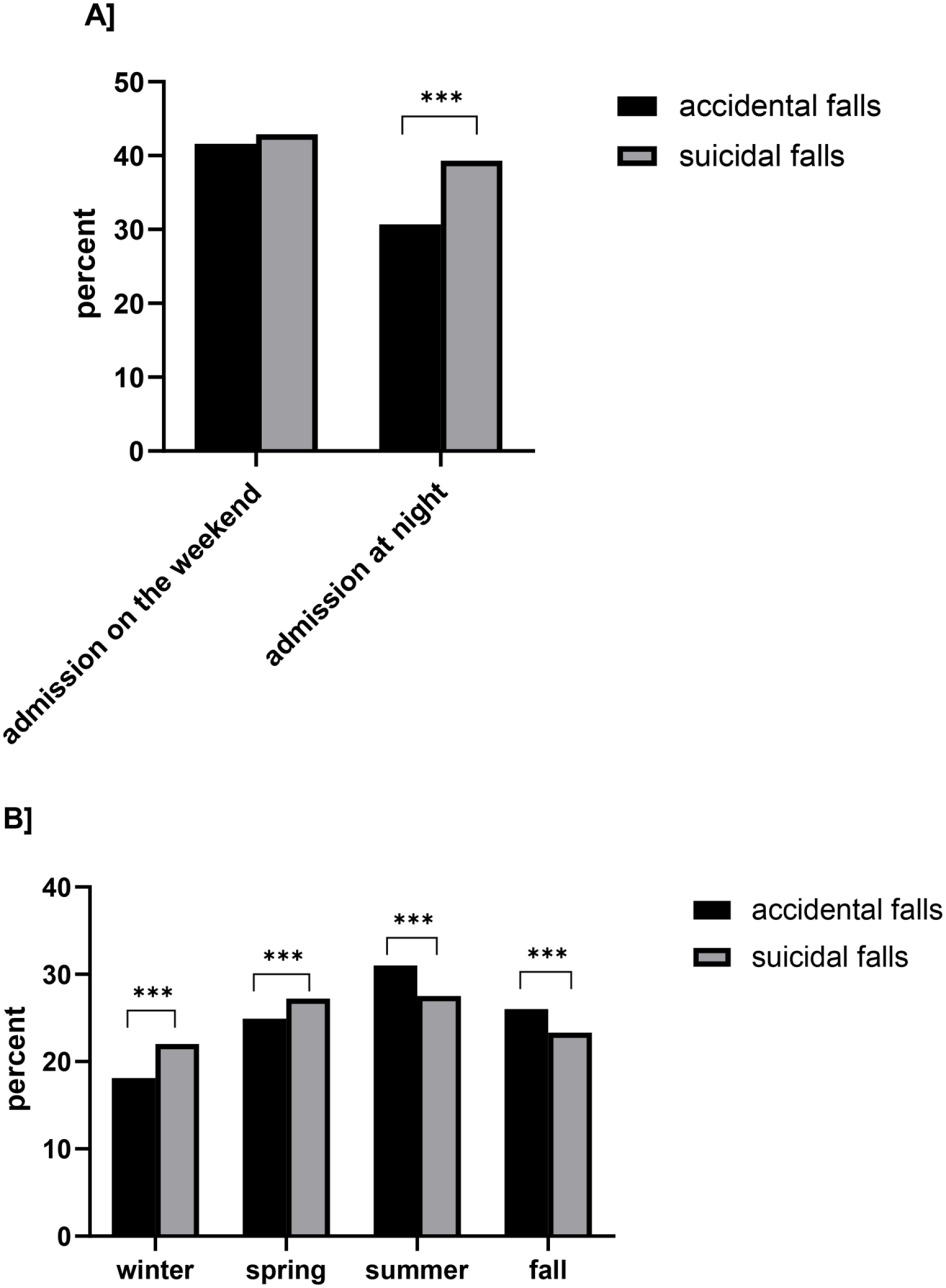
Comparison of admission times for accidental and suicidal falls. **A**: Comparison between admission on the weekend and admission at night. **B**: Comparison of by season. The data are presented as percentages of the respective group involved

Regarding the timing of the incidents, it was found that attempted suicidal falls occurred somewhat more frequently at night (39.3%, *n* = 2,085; *p* < 0.001) as well as in winter (22.0%, *n* = 1,166; *p* < 0.001) and spring (27.2%, *n* = 1,442; *p* < 0.001), whereas accidents were less common at night (30.7%, *n* = 7,575; *p* < 0.001) and more frequently in summer (31.0%, *n* = 7,656; *p* < 0.001) and autumn (26.0%, *n* = 6,409; *p* < 0.001, Fig. 4a and b).

**Fig. 5:**
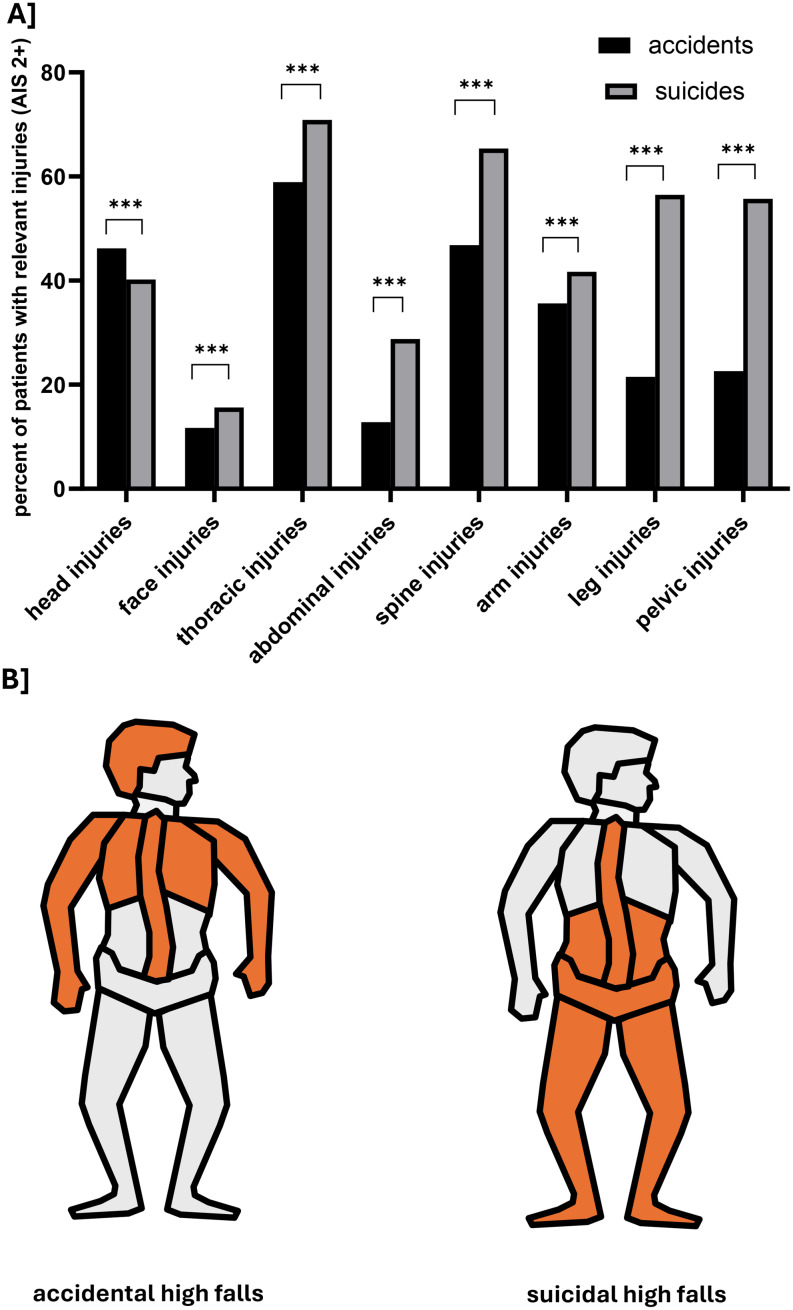
Comparison of injury pattern from accidental and suicidal high falls. **A**: Direct comparisons of the frequency of the injured body parts. The data are presented as percentages of the respective group involved. ***=*p* < 0.001. **B**: Schematic illustration of the four most common injured body parts

The analysis of injury patterns revealed differences between accidents and suspected suicides within the high falls group: Accidents most frequently involved injuries to the thorax (58.9%), spine (46.8%), head (46.2%), and arms (35.6%). Suicides, on the other hand, more often resulted in multiple injuries, with the most common being to the thorax (70.9%), spine (65.4%), legs (56.5%), and pelvis (55.7%). These findings suggest that individuals injured in accidents are more likely to land headfirst, whereas suicides more often land feet first (Fig. 5a and b). An injury to the respective body region was counted if it had an Abbreviated Injury Scale (AIS) score of 2 or higher [8].

### Prehospital and resuscitation room management of high falls

We also examined differences in prehospital and early clinical management of various types of falls, as this early phase can be particularly relevant for outcomes and may allow for the development of specific recommendations. The previously described higher injury severity of suicidal falls is also reflected here: In this group, significantly more prehospital intubations occurred at 39.6% compared to 21.5% in accidental falls (*p* < 0.001). Resuscitation itself was also significantly more common in the suicidal group at 10.0% compared to 2.7% in accidental falls (*p* < 0.001). Accordingly, significantly more red blood cell concentrates were administered in the resuscitation room for suicidal falls (29.9% vs. 6.6% for accidental falls, *p* < 0.001). Emergency surgeries in the resuscitation room were also significantly more frequent in suicidal falls at 46.5% compared to 24.6% in accidental falls (*p* < 0.001).

After initial management, patients with suicidal falls spent an average of 11 days (IQR 5–22) in the ICU and 24 days (IQR 14–39) in total in the hospital, compared to 2 days (IQR 1–7) in the ICU (*p* < 0.001) and 13 days (IQR 8–22) in the hospital (*p* < 0.001) for accidental falls. Interestingly, the time from the incident to hospital arrival was longer for accidental falls, with a median of 62 min (IQR 49–80) compared to 55 min (IQR 42–72) for suicidal falls (*p* < 0.001; Table 1).

### Workplace accidents within the high falls group

Since 2020, the TraumaRegister DGU^®^ has also recorded whether the incident was a workplace accident. Among high fall accidents included since 2020, 20.3% were workplace-related (*n* = 2,043). The mean age of work-related accident victims was 46.6 years (SD 14.9), with an ISS of 21.8 (SD 11.6). In 96.3% of these accidents, men were affected. Interestingly, despite a comparable ISS, the in-hospital mortality rate for workplace-related falls is significantly lower at 6.5% compared to 16.1% of non-occupational falls (Table 2).

**Fig. 6:**
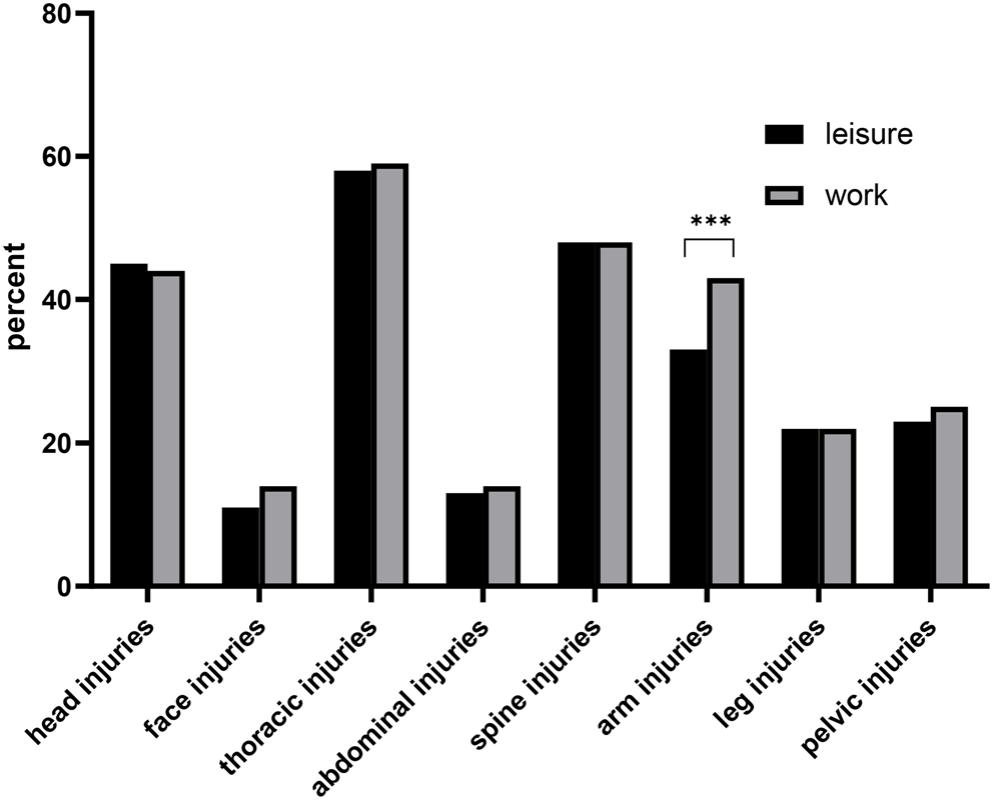
Comparison of injury pattern from leisure-related and work-related high falls. **A**: Direct comparisons of the frequency of the injured body parts. The data are presented as percentages of the respective group involved

The distribution of injury patterns was relatively similar between work-related and leisure-related high-falls. However, work-related falls showed a slightly higher proportion of arm injuries (43.0% vs. 33.0%, *p* < 0.001; Fig. 6).

**Table 2:** Basic data of high falls, available since 2020

High falls	Leisure-related (*n* = 8,001)		Work-related (*n* = 2,043)	
***General data***				
Age (mean)	55.0	SD 21.6	46.6	SD 14.9
ISS (mean)	23.3	SD 12.4	21.8	SD 11.6
Male sex (n)	5893	73.7%	1967	96.3%
***Prehospital / Emergency room***				
Prehospital time (minutes, median)	64	IQR 50–81	65	IQR 53–80
Resuscitation (n)	346	4.5%	53	2.6%
Intubation (n)	1,836	22.9%	426	20.9%
Emergency surgery (n)	2,324	29.0%	592	30.0%
Blood transfusion (n)	1,026	12.8%	147	7.2%
***Trauma care level***				
Supra-regional (n)	5,352	66.9%	1,515	74.2%
Regional (n)	2,116	26.5%	452	22.1%
Local (n)	533	6.7%	76	3.7%
***Clinical course***				
ICU Length of stay (days, median)	3	IQR 1–10	2	IQR 1–6
Hospital Length of stay (days, median)	12	IQR 6–22	11	IQR 6–20
Died in hospital (n)	1,290	16.1%	133	6.5%
Died within 24 h (n)	721	9.0%	72	3.5%

SD: Standard deviation; IQR: Interquartile range

## Discussion

Falls from great heights continue to represent a significant portion of the orthopedic/trauma surgery patient population and are associated with considerable mortality. To date, there is very limited knowledge regarding potential differences in the timing of the accident, injury patterns, and clinical outcomes between various types of falls. Consequently, no tailored recommendations for action can be derived during the early clinical phase.

In this study, we retrospectively analyzed data since 2014 on falls from great heights (> 3 m) and low heights (< 3 m). Additionally, falls from great heights were further categorized based on their circumstances into suicidal and accidental, with accidental falls subdivided into occupational and non-occupational. To our knowledge, we are the first to conduct this detailed categorization of high falls in such a large patient cohort, aiming to identify relevant differences that could prove valuable for clinical practice, particularly during the initial treatment phase.

As expected, the high falls group, with an average age of approximately 52 years, is significantly younger than the low falls group, which has an average age of nearly 70 years. Thus, the low falls group predominantly consists of a fall-prone geriatric patient population. Appropriately, the ISS following a fall in these older patients is lower at 18.7 compared to 23.0 in the high falls group. Interestingly, however, low falls in these geriatric patients still represent a serious event: Following a low fall, a significantly higher percentage of patients (22.6%) died compared to 13.6% after a high fall. Notably, over half of the low falls involved head injuries. Older patients often undergo long-term anticoagulation therapy, which significantly increases the risk of intracerebral hemorrhages even after mild head injuries [9, 10]. These findings emphasize that falls among geriatric patients should be taken seriously, as they are associated with high morbidity and mortality [11]. Often, a predisposition to falling exists long before a fall event necessitating hospitalization occurs. Therefore, a tendency to fall in older patients should prompt the consistent implementation of preventive measures [12, 13].

In early clinical management, it appears advisable to initiate comprehensive diagnostics, even when patients with impaired cognitive performance may not adequately report their symptoms. Particular attention should be given to detecting head injuries.

When comparing accidental falls and suicidal falls, it is noticeable that suicidal patients are, on average, about 46 years old—approximately 7 years younger. This age is about 10 years below the general peak age for suicides in Germany [14]. While accidents predominantly affect men, the gender ratio in suicides is roughly balanced. It is worth noting that patients who experienced a suicidal fall were significantly more severely injured and died more than twice as often in the hospital, which has also been reported in other non-European studies [15, 16]. Kang and colleagues attributed this, among other factors, to a greater fall height in suicides. Correspondingly, these patients required significantly more prehospital intubations, resuscitations, blood transfusions, and emergency surgeries.

In our data, roughly 18% of high falls are suicidal attempts. The proportion of suicide attempts among high falls is inconsistently reported in the literature, ranging between 13% and 50%, likely due to the often unclear cause at the initial assessment [7, 17].

The injury patterns of the two types of falls differ markedly: suicidal falls are significantly more likely to result in extremity and pelvic trauma, while accidental falls more often lead to head injuries. Suicidal falls therefore frequently appear to involve a “feet-first” mechanism, whereas accidental falls occur in a more uncontrolled manner [18]. Accordingly, suicidal falls show a predominantly caudal injury distribution, while accidental falls are characterized by a cranial injury predominance. Regarding the time of day and season, the data on general suicide rates remain inconclusive, without a clear trend [19–21]. However, we observed that suicidal falls, in comparison to accidental falls, occur slightly more often at night and more frequently during winter and spring. Notably, despite reports of higher overall suicide rates during winter in epidemiological studies, suicidal falls were least frequent in winter in our cohort compared to other seasons. This discrepancy may indicate that during winter months other suicide methods are preferentially used, while falls from height contribute less to overall suicide incidence.

For early clinical management, this suggests that it is worthwhile to attempt to determine the circumstances of a fall as soon as a high-fall trauma patient is announced. If a suicidal intent is suspected, the treating team should be instructed to prepare for potential emergency surgeries, intubations, and blood transfusions, and to streamline the corresponding workflows. Special attention should be paid to pelvic stabilization and thorough diagnostics of the distal lower extremities in suicidal falls.

Work-related falls primarily affect men, likely due to the fact that many high-risk occupations, such as construction work, continue to be predominantly male. Interestingly, work-related falls have a comparable ISS to non-work-related accidents, but significantly fewer patients die in the hospital (6.5% compared to 16.1% in leisure-related falls). One possible explanation is that workplace falls are more often witnessed and better medical infrastructure is available, enabling faster initiation of medical measures.

The injury patterns of leisure-related and work-related high falls are comparable. Only the arms are slightly more frequently affected in work-related high falls. One possible explanation for this is that falls occur more often during manual tasks, possibly leading to the involvement of the arms. Another possibility is that in some cases, arm injuries occurred first, which then led to a fall.

This study is not without limitations. First, it is a retrospective analysis with the well-known inherent limitations. Additionally, the TraumaRegister DGU^®^ data only allow differentiation between high falls (> 3 m) and low falls (< 3 m). Further differentiation of fall heights is not possible but could be valuable for a more nuanced risk adjustment of the fall types. Furthermore, only data from patients who survived the prehospital phase after a fall are captured, meaning potential biases in the reported injury patterns cannot be ruled out. Moreover, these findings therefore provide limited recommendations for the prehospital phase. Finally, the registry does not provide detailed information on pre-existing comorbidities or acute intoxications, which may represent important confounders influencing both fall mechanism and injury patterns.

In conclusion, there are characteristic differences between various types of falls. Low falls frequently affect geriatric patients, who may still suffer severe injuries and have high mortality rates. High falls resulting from suicide attempts involve more severe injuries and higher mortality rates than accidents. Additionally, suicidal falls are more likely to involve extremity and pelvic injuries, whereas accidents are more commonly associated with head injuries. Work-related accidents are comparable to non-work-related accidents in principle but have lower mortality rates, possibly due to faster access to medical care or due to occupational safety measures.

## Data Availability

The data set used and analyzed during the current study is not available due to the data protection guidelines of the German Trauma Society.
